# Comparing harm reduction and overdose response services between community-based and public health department syringe service programmes using a national cross-sectional survey

**DOI:** 10.1016/j.lana.2024.100757

**Published:** 2024-05-06

**Authors:** Bradley R. Ray, Jamie L. Humphrey, Sheila V. Patel, Christopher F. Akiba, Ricky N. Bluthenthal, Hansel Tookes, Paul A. LaKosky, Lynn D. Wenger, Alex H. Kral, Barrot H. Lambdin

**Affiliations:** a RTI International, Research Triangle Park, NC, United States; b University of Southern California, Los Angeles, CA, United States; c University of Miami Miller School of Medicine, Miami, FL, United States; d North American Syringe Exchange Network, Tacoma, WA, United States

**Keywords:** Syringe service program, Harm reduction, Community-based organization, Public health

## Abstract

**Background:**

Syringe services programmes (SSPs) are an evidence-based strategy to reduce infectious diseases and deliver overdose prevention interventions for people who use drugs. They face regulatory, administrative, and funding barriers that limit their implementation in the US, though the federal government recently began providing funding to support these efforts. In this study we aim to understand whether the organisational characteristics of SSPs are associated with the provision of syringe and other overdose response strategies.

**Methods:**

We examine four outcomes using the National Survey of Syringe Services Programs (NSSSP) (N = 472): syringe distribution, naloxone distribution, fentanyl test strip (FTS) availability, and buprenorphine implementation. These outcomes are assessed across three organizational categories of SSPs—those operated by public health departments (DPH), community-based organizations (CBOs) with government funding, and CBOs without government funding—while adjusting for community-level confounders.

**Findings:**

The proportion of SSPs by organizational category was 36% DPH, 42% CBOs with government funding, and 22% CBOs without government funding. Adjusting for community-level differences, we found that CBO SSPs with government funding had significantly higher provision of all four syringe and overdose response services as compared to DPH SSPs and across three of the four services as compared to CBO SSPs without government funding. CBO SSPs without government funding still had significantly higher provision of three of the four services as compared to programmes maintained by the DPH.

**Interpretation:**

CBO SSPs have strong potential to expand overdose response services nationally, particularly if provided with sustained and adequate funding. Communities should aim to provide funding that does not hinder SSP innovation so they can remain flexible in responding to local needs.

**Funding:**

This study was supported by 10.13039/100014848Arnold Ventures (20-05172).

**Research in context**

**Evidence before this study**

Researchers and practitioners have long stressed the importance of harm reduction as an evidence-based service delivery approach and public health strategy for reducing drug related harms, particularly the spread of infectious disease. In the United States, these harm reduction services and strategies have largely been disseminated through syringe service programmes, which provide a variety of health care needs for persons who use drugs. However, there is a paucity of information on the influence of organizational characteristics among these programmes and the provision of harm reduction and overdose prevention practices. We searched Pubmed, Medline using the search strategy “syringe service programme” and “funding” and “non-profit” and “public health department” through August 2023. Prior research in the Americas has demonstrated effectiveness of syringe service programmes in reducing infectious disease, cost effectiveness, and the impact of state laws. However, there is a paucity of information on the influence of organizational characteristics among these programmes and the provision of harm reduction and overdose prevention practices.

**Added value of this study**

This study is the first to examine the association between syringe service programme characteristics and output. Using cross-sectional data from the National Survey of Syringe Services Programs we created three mutually exclusive types of programmes: those operated by public health departments, community-based organizations with government funding, and community-based organizations without government funding. Adjusting for community-level confounders we examined syringe distribution, naloxone distribution, fentanyl test strip availability, and buprenorphine implementation to find that community-based syringe service programmes with funding far outpace those in public health departments in the provision of harm reduction and overdose prevention materials.

**Implications of all the available evidence**

Harm reduction has recently received federal support and it is important that research inform effective delivery of lifesaving public health programming. Syringe service programmes have become multipurposed, providing resources for a variety of healthcare needs, and the history of these efforts is rooted in community-led responses from, and for, persons who use drugs. While further research is needed to understand the causal mechanism, this study suggests that community-based syringe service programmes have strong potential for expanding services nationally if they are provided with sustained and adequate funding.

## Introduction

For more than 30 years syringe services programmes (SSPs) have been a critical part of the United States public health strategy towards reducing HIV and blood-borne infection transmission through distributing new syringes and promoting safer injection.^1^ There is considerable research showing SSPs can be effective at reducing infections among people who inject drugs and provide cost-savings.^2^, ^3^, ^4^ Harm reduction strategies like SSPs focus on meeting people where they are, even when they are not interested, ready, or able to stop using substances; however, many people do not have access to these services because of legal, regulatory, and funding barriers that stem from stigma and criminalisation of drug use.^5^ Despite ample evidence of effectiveness, federal funds have traditionally excluded SSPs and, in particular, have prohibited the use of federal funds to purchase of syringes and other safer drug use supplies.^6^

The overdose mortality epidemic is now driving decreased national life expectancy rates and continues to be one of the most pressing public health problems in the United States.^7^ Across multiple waves—from prescription opioids, to heroin, to illicitly manufactured fentanyl—overdose rates have continued to increase, with recent trends showing mortality highest among Black, Indigenous and people of colour and adolescents.^8^ With the continued significance of opioids in the overdose epidemic, additional response strategies have emerged within syringe service programming. For example, while SSPs have long been champions for community-based naloxone distribution (the medication that reverses opioid overdoses), some now accelerate these efforts through strategies like vending machines and mail programmes.^9^^,^^10^ Fentanyl test strips (FTS) are an emerging strategy used by SSPs; given the proliferation of fentanyl (a synthetic opioid 50 to 100 times more powerful than heroin) in heroin, but especially throughout the illicit drug supply including stimulants and counterfeit pills, paper test strips and other drug checking have emerged as a potential overdose mortality prevention strategy.^11^ Drug checking can include testing for the type and quantity of substances; but, more often, and especially given the ability to use federal funding, it includes the distribution of FTS which are small strips of paper that detect the presence of fentanyl with similar strips developed for xylazine.^12^ Finally, SSPs are also playing a role in expanding access to evidence-based medications for opioid use disorder; while national experts continue calling for easier access to methadone, it remains highly regulated, making buprenorphine the primary medication for SSPs aiming to provide immediate low-barrier pathways to treatment.^13^

Without legal sanction or funding in the United States, early SSP implementation efforts were built as mutual aid collectives by committed volunteers; and even today, many programmes are operated entirely by volunteers.^14^, ^15^, ^16^ As states passed legislation that authorised SSPs to operate, early volunteer-based SSPs transformed into non-profit, community-based organizations. In addition, some county and state health departments began considering how to support SSP implementation either through funding or by establishing SSPs operated by departments of public health.^17^^,^^18^ Attributes of these organizational types and resources can have substantial implications for service delivery; to date, no empirical investigations have sought to understand the impact of organizational types and funding on SSP service delivery.^19^

In this study, we seek to learn whether the organizational type and funding of SSPs is associated with needs-based distribution of these harm reduction and overdose response services. Using data from a national survey of SSPs, we categorise organisational context as those operated by a public health department (DPH) versus a community-based organization (CBO) and further categorise the CBOs as those with and without any government source of funding. Across these three SSP organisation categories—(1) *DPH SSP, (2) CBO SSP with government funding, (3) CBO SSP without government* funding—we examine syringe and naloxone distribution along with availability of on-site buprenorphine and FTS, while adjusting for community level factors that might influence overdose prevention strategies at a SSP; urbanicity, overdose mortality rate, and voting patterns, the latter of which has been associated with disease prevention efforts including the presence of a SSP.^20^

## Methods

### Study design and procedures

Supported by Arnold Ventures, scientists at RTI International, in collaboration with the North American Syringe Exchange Network (NASEN), carried out annual, cross-sectional surveys of syringe services programmes, referred to as the National Survey of Syringe Services Programs (NSSSP) in 2021 and 2022. The NSSSP was originally designed to understand the impact of state-level policy initiatives on services delivered from SSPs. To carry out the NSSSP, we invited all SSPs to participate by emailing organizational directors from a database of SSPs known to be operating in the United States. This SSP database was built by proactively searching and contacting SSPs from several sources, including the North American Syringe Exchange Network (NASEN) Buyers’ Club and online directory; social media platforms; SSP networks; conferences; webinars; as well as state and county DPH websites. For this analysis, data were included from the 2021 and 2022 NSSSP surveys, which have been detailed previously.^21^, ^22^, ^23^ Regarding NSSSP inclusion criteria, SSPs had to be operational and were defined as organizations focused on engaging PWUD and providing drug use supplies, such as sterile syringes and injection-related equipment, to reduce harms associated with drug use. SSPs frequently provide a variety of other services to improve the health of people who use drugs, such as overdose education and naloxone distribution; drug checking; vaccinations; screening and linkage to medical care; substance use treatment; and other social support services.^24^

Organisational directors of SSPs were emailed up to three times asking them or their designee to participate in the online survey (Voxco©, Montreal, Quebec, Canada); for non-responding SSPs, we conducted individual follow-up via email and phone calls. SSPs were offered a $75 honorarium if they completed the survey. Included in this analysis, NSSSP 2021 and NSSSP 2022 were administered from February–July 2021 and February–July 2022, achieving a 64% (307/481) and 69% (305/444) response rate, respectively. Our analytic plan adjusts for repeated measures among programmes that participated in both surveys. The NSSSP data were geocoded to 2020 Census Bureau cartographic county boundaries to represent the county where the SSP is headquartered to include community-level confounding measures as described below. RTI International’s institutional review board within the Office of Research Protection reviewed and approved these survey procedures (MOD00001166).

### Outcomes and measures

We include four outcome measures from the NSSSP to examine harm reduction and overdose response services: (1) count of participant contacts where syringes were distributed, (2) count of participant contacts where naloxone was distributed, (3) FTS implementation (yes/no), and (4) buprenorphine implementation (yes/no). Syringe and naloxone contacts are a continuous measure (“How many participant contacts for *syringe services*/*providing naloxone* occurred at your SSP in *2021/2020*?”) while buprenorphine and FTS were measured as dichotomous based on availability (1 = yes). For buprenorphine, SSPs indicated whether they had implemented the services online or in-person in the previous year (“Were on-site *and/or* virtual medical services for buprenorphine/suboxone available to your participants in 2021”). FTS distribution was based on the survey item “How many fentanyl test strips did your SSP provide to participants in 2021?”; however, because of differences in how strips were counted we used an indicator measure where SSPs were assigned a value of ‘1’ if they provided at least 1 FTS in the previous year, and ‘0’ if no strips were distributed.

Our exposure measure was based on SSP organizational types in combination with the funding source item from the survey (“What were your syringe service programme’s sources of funding for the last fiscal or calendar year?”). The categories included: (1) DPH, (2) CBO with government funding, and (3) CBO without government funding. The DPH category refers to any SSP that is part of a city, county, or state government while CBOs refer to any standalone non-profit organization, any SSP embedded within a larger non-profit organization, and those unfunded. There were no commercial (for profit) SSPs. CBO SSPs without government funding primarily reported donations as the primary funding source while those with funding indicated state more frequently than federal sources.

Potential community-level confounders include urbanicity, overdose mortality rate, and voting patterns in the 2020 presidential election. We constructed a three-tier, county-level measure of urbanicity from the 2013 NCHS Urban-Rural Classification Scheme^25^ to account for geographic variation in access to SSPs.^26^ Leveraging data from a previous study,^27^ we used smoothed, county-level opioid overdose mortality rates from 2019 as a marker of a community’s need for overdose response services. Finally, voting patterns came from the MIT Election Science Lab^28^ and reflected percent of county residents that voted for a republican candidate in the 2020 presidential election from the total number of votes. There is an ongoing debate about pathways for Republican presidential vote share to contribute to the poorer health outcomes.^29^ This measure may represent county-level norms for using public resources, support for harm reduction, and/or economic distress.^30^^,^^31^

### Statistical analysis

We used negative binomial generalised estimating equations (GEE) to assess the impact of organisational categories (DPH SSP, CBO SSP with government funding, CBO SSP without government funding) on the two continuous outcomes (syringe contacts and naloxone contacts) with each scaled to represent 1000 persons and results presented as adjusted incident rate ratios (aIRR) with 95% confidence intervals (CI). For dichotomous outcomes (buprenorphine implementation and FTS implementation), we used logit GEEs with results presented as adjusted odds ratios (aOR) and 95% CI. For each GEE model, DPH SSP was used as the reference category for the 3-level exposure variable, rural was used as the reference category for urbanicity, and two continuous measures (overdose mortality rate and percentage republican votes) were included as potential confounders. Each continuous measure was standardised with a mean = 0 and standard deviation (SD) = 1. We used an exchangeable correlation matrix to account for SSPs clustered within survey years.^32^ All variables with a p < 0.05 were considered significant. Data preparation and analyses were conducted in SAS Enterprise Guide version 7.15. All STROBE checklist items are included for cross-sectional studies.

### Role of the funding source

This study was supported by Arnold Ventures, Contract No. 20-05172, to Dr. Barrot H. Lambdin (PI). They had no role in the design, data collection, analysis, interpretation of results, writing of the manuscript, or decision to submit for publication as researchers maintained full independence with the content solely reflecting the views of the authors.

## Results

In terms of SSP organisational context, 36% (N = 169) of SSPs were DPH, while the remaining 64% were CBOs (42% were CBOs with government funding and 22% were CBOs without government funding) (Table 1). In looking at outcomes (Table 2) the median number of syringe and naloxone contacts by SSPs was 1176 (Interquartile range [IQR] = 300–3575) and 472 (IQR = 133–1536), respectively. Syringe and naloxone contacts were highest in CBOs with government funding (syringe: Median = 2373; IQR = 800–6524; naloxone: Median = 1026; IQR = 404–3000). Syringe contacts were lowest in CBOs without government funding (Median = 530; IQR = 150–2000); Naloxone contacts were lowest in DPH (Median = 195; IQR = 27–673). We found that 73% of SSPs provided FTS and 34% provided buprenorphine; CBO SSPs with government funding were most likely to provide FTS (54%) and buprenorphine (65%), with similar availability across the SSPs operated by a DPH or CBOs without government funding (FTS: 23%; buprenorphine: N = 18%).

**Table 1:** Descriptive statistics of binary or categorical syringe services program characteristics and county-level measures in the 2021 and 2022 National Survey of Syringe Service Programs (N = 472).

	N	%
Syringe services program characteristics		
SSP organizational category x government funding		
DPH	169	35.8
CBO–Gov Funding	200	42.4
CBO–No Gov Funding	103	21.8
FTS availability		
Overall	342	72.5
DPH	79	23.1
CBO–Gov Funding	184	53.8
CBO–No Gov Funding	79	23.1
Buprenorphine availability		
Overall	158	33.5
DPH	28	17.7
CBO–Gov Funding	102	64.6
CBO–No Gov Funding	28	17.7
County-level characteristics		
Urbanicity		
Rural	107	22.7
DPH	75	70.1
CBO–Gov Funding	18	16.8
CBO–No Gov Funding	14	13.1
Suburban	240	50.9
DPH	80	33.3
CBO–Gov Funding	104	43.3
CBO–No Gov Funding	56	23.3
Urban	125	26.5
DPH	14	11.2
CBO–Gov Funding	78	62.4
CBO–No Gov Funding	33	26.4

IQR = interquartile range; SSP = syringe services program; DPH = SSP run by a department of public health; CBO—Gov Funding = community-based organization *with* governmental funding; CBO–No Gov Funding = community-based organization *without* governmental funding.

**Table 2:** Descriptive statistics of continuous syringe services program characteristics and county-level measures in the 2021 and 2022 National Survey of Syringe Service Programs (N = 472).

	N	Median	IQR
Syringe services program characteristics			
Syringe contacts			
Overall	472	1176	(300–3575)
DPH	169	630	(141–2054)
CBO–Gov Funding	200	2373	(800–6524)
CBO–No Gov Funding	103	530	(150–2000)
Naloxone contacts			
Overall	472	500	(133–1536)
DPH	169	195	(27–673)
CBO–Gov Funding	200	1026	(404–3000)
CBO–No Gov Funding	103	350	(147–1404)
County-level characteristics			
Opioid overdose mortality rate per 100k, 2019			
Overall	472	14.1	(9.5–24.5)
DPH	169	13.9	(9.9–23.9)
CBO–Gov Funding	200	15.4	(9.6–25.4)
CBO–No Gov Funding	103	12.7	(8.1–22.3)
% Voted Republican, 2020			
Overall	472	39.5	(24.0–50.4)
DPH	169	44.4	(30.9–63.9)
CBO–Gov Funding	200	35.5	(20.5–45.1)
CBO–No Gov Funding	103	41.0	(24.0–49.4)

IQR = interquartile range; SSP = syringe services program; DPH = SSP run by a department of public health; CBO—Gov Funding = community-based organization *with* governmental funding; CBO–No Gov Funding = community-based organization *without* governmental funding.

We include descriptive statistics from community-level control measures in Tables 1 and 2 as well. Median opioid overdose mortality rates were similar across SSP organization categories with an overall median rate of 14.1 per 100,000 county population (IQR = 9.5–25.4). Median percent of republican voters was highest in counties where SSPs were operated by a DPH (44%; IQR = 31–64%) and the lowest in counties where SSPs were CBOs with government funding (36%; IQR = 21–45%). Overall, SSPs were most common in suburban counties (51%) followed by urban (26%) and rural counties (23.0%). Different types of SSPs were operational in 6% of counties included in this study. Data were missing for key variables for 140 SSPs, creating an analytic sample of n = 472 SSPs. Sensitivity analyses found that variables in Tables 1 and 2 were not meaningfully different between the full (n = 612) and analytic (n = 472) sample.

**Fig. 1:**
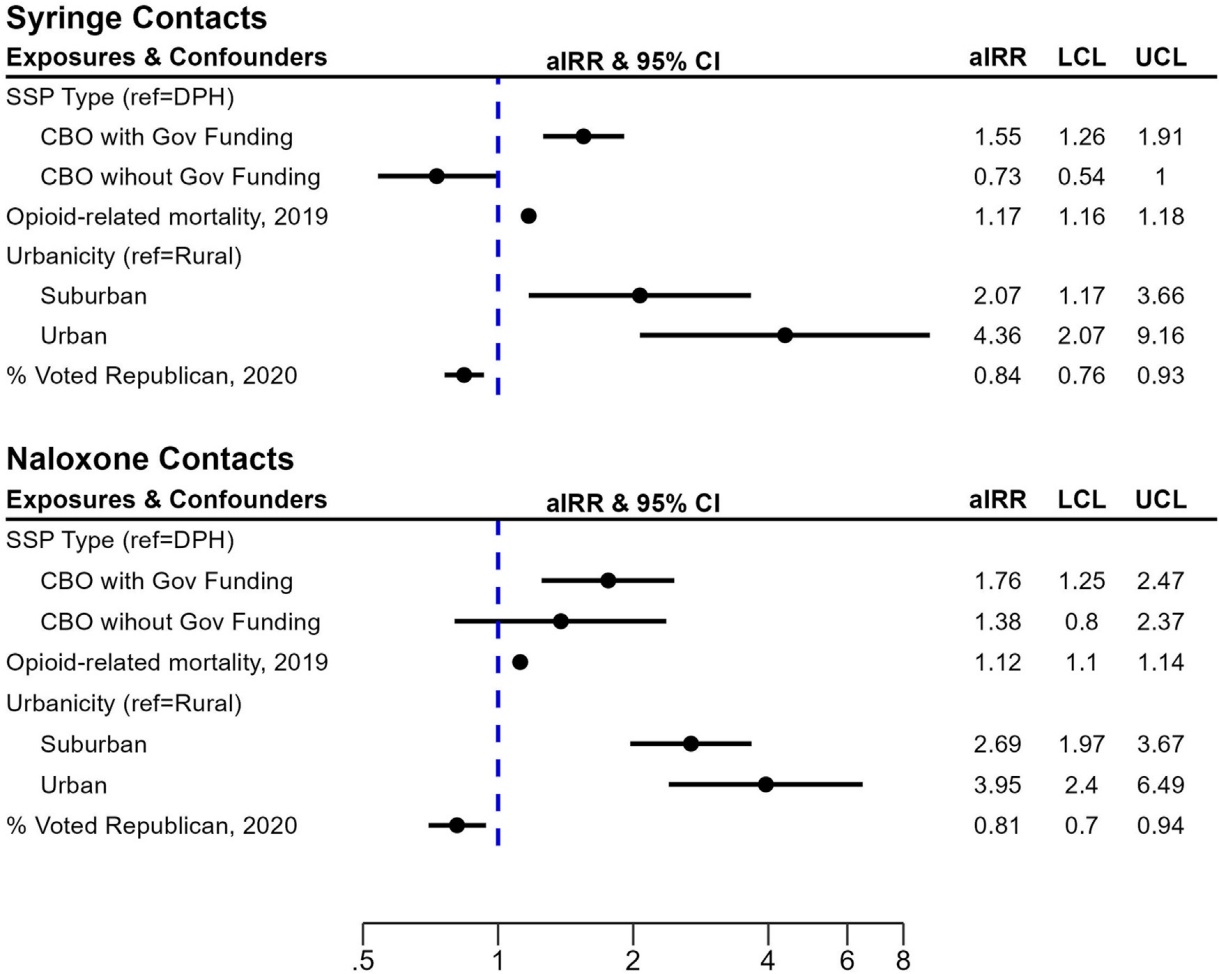
Forest plots of results from negative binomial GEE models (N = 472). SSP = syringe service program; GOV = governmental SSP; NGO—Gov Funding = non-governmental *with* governmental funding; NGO–No Gov Funding = non-governmental *without* governmental funding; aIRR = adjusted Incident Rate Ratio; CI = confidence interval; LCL = lower confidence limit; UCL = upper confidence limit.

Negative binomial GEE models showed that syringe and naloxone contacts were higher among CBO SSPs with government funding as compared to DPH SSPs (Fig. 1). Syringe contacts were 55% higher among CBO SSPs with government funding (aIRR = 1.55, 95% CI = 1.26–1.91) than DPH SSPs which are statistically similar to CBO SSPs without government funding (Fig. 1). Similarly, naloxone contacts were 1.76 (95% CI = 1.25–2.47) times higher among CBO SSPs with government funding, relative to DPH SSPs; naloxone contacts distributed by CBO SSPs without government funding were not statistically different than DPH SSPs. For both outcomes, the pattern and effect size across covariates was similar; for example, a standard deviation increase in county opioid overdose mortality rate was associated with a 17% or 12% increase in syringe and naloxone contacts [aIRR = 1.17, 95% CI = 1.16–1.18; aIRR = 1.12, 95% CI = 1.10–1.14, respectively]. Syringe and naloxone contacts were significantly higher in urban [aIRR = 4.36, 95% CI = 2.07–9.16; aIRR = 3.95, 95% CI = 2.40–6.49, respectively] and suburban counties [aIRR = 2.07, 95% CI = 1.17–3.66; aIRR = 2.69, 95% CI = 1.97–3.67, respectively], relative to rural counties. Additionally, an increase in republican voting was associated with decreased syringe and naloxone contacts [aIRR = 0.84, 95% CI = 0.76–0.93; aIRR = 0.81, 95% CI = 0.70–0.94, respectively].

**Fig. 2:**
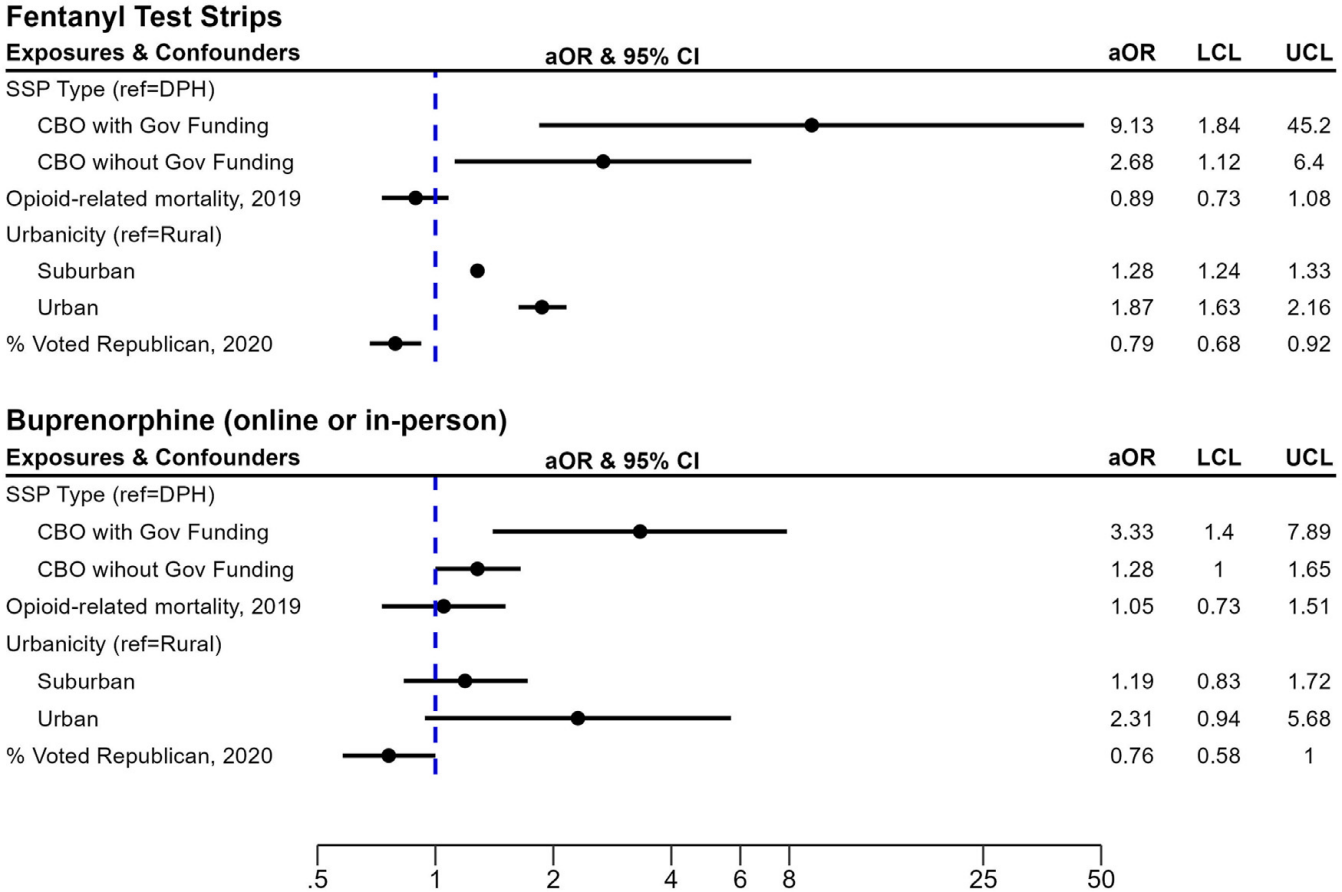
Forest plots of results from Logit GEE models (N = 472). SSP = syringe service program; GOV = governmental SSP; NGO—Gov Funding = non-governmental *with* governmental funding; NGO–No Gov Funding = non-governmental *without* governmental funding; aOR = adjusted odds ratio; CI = confidence interval; LCL = lower confidence limit; UCL = upper confidence limit.

Results from logit models shows FTS and buprenorphine implementation were also significantly higher among CBO SSPs with government funding relative to DPH SSPs (Fig. 2). The odds of FTS were 9.13 (95% CI = 1.84–45.20) and 2.68 (95% CI = 1.12–6.40) times higher in CBO SSPs with and without government funding, respectively, compared to DPH SSPs (Fig. 2) with similar results for buprenorphine. Higher rates of republican voting are associated with decreased odds of FTS distribution (aOR = 0.79, 95% CI = 0.68–0.92) and buprenorphine (aOR = 0.76, 95% CI = 0.58–1.00). Odds of FTS distribution was significantly higher in urban (aOR = 1.87, 95% CI = 1.63–2.16) and suburban counties (aOR = 1.28, 95% CI = 1.24–1.33), relative to rural counties; urbanicity was not significantly associated with buprenorphine. County-level opioid mortality rate was not significantly associated with either outcome.

## Discussion

Results from our analysis of a national survey of SSPs in the United States shows that programmes implemented by a DPH provide fewer harm reduction and overdose response services than CBO SSPs. Looking across multiple outcomes—the number of contacts receiving resources like syringes and naloxone, but also the availability of newer resources, such as FTS and buprenorphine—we found that our organisational categories were an important consideration in the distribution of these services. SSPs implemented by a CBO with government funding provided more services than DPH and CBO SSPs without government funding. While it was beyond the scope of this study to measure community demand or participant acceptability, we attempted to adjust for community level factors that could impact SSP implementation and practices: urbanicity, overdose mortality rate, and political voting patterns. Even after controlling for these factors, CBOs with government funding had significantly higher syringe contacts, almost twice the naloxone contacts, and nine times higher odds of providing FTS and three times higher odds of providing buprenorphine than DPH SSPs.

This study provides important insight into the association between organisational context and the provision of harm reduction strategies among SSPs. There are numerous examples of health departments supporting the facilitation of CBO SSPs, including the implementation of emerging harm reduction strategies.^13^ Yet, these efforts often occur against the backdrop of legal concerns that could serve as a greater barrier among DPH SSPs. For example, with the emergence of paper drug checking test strips, DPH SSPs might face restrictions for purchasing or distributing these test strips if they violate local or state paraphernalia laws.^26^ Thus, our findings might reflect systemic barriers impeding the provision of harm reduction resources by DPH SSPs. However, as other studies suggest, the flexibility of CBO SSPs might enable uptake of innovative service delivery practices.^15^ For example, following the COVID-19 pandemic, when the federal government waived in-person examination for initial consultation for buprenorphine treatment, SSPs started using telehealth to provide immediate linkage to care.^15^ Alternatively, CBO SSPs could also have greater access to an existing social network of persons using drugs, and thereby, be better able to understand and adapt to changing community needs.

More research is needed to understand the mechanism of action, and this should include a consideration of local policies. For example, we found DPH SSPs were located in counties with higher levels of Republican voting, which likely reflects policy barriers to implementing CBO SSPs.^20^ For example, SSPs located in Kentucky and Ohio, states overwhelmingly represented by Republican votes in the 2020 presidential election, are only eligible to be operated by the local health departments.^33^ Similarly, in Indiana—a state that experienced dire public health consequences from a lack of SSPs in 2015 with an HIV outbreak^34^—a physician, physician assistant, or registered nurse must provide oversight of the SSP, yet it remains a felony to possess a syringe without a prescription.^35^ This results in approved county health departments distributing syringes, while police undermine those evidence-based public health efforts by arresting people for possessing syringes and other harm reduction supplies.^36^, ^37^, ^38^ The results from this study might also be driven by urbanicity, where there could be greater resources or need for non-profits to fill service gaps, and the population density of these urban centres might also explain trends in syringe and naloxone distribution.

While this study has clear public health implications it is not without limitations. Data were derived from self-report cross sectional survey data. As such, potential recall bias exists which is common for organisational surveys of this nature. Additionally, this analysis combined two serial cross-sectional surveys where one organisation is likely to have completed the survey in both years, potentially affecting the standard errors. We believe the lack of independence across survey years is unlikely to have impacted our findings since we employed an analytic strategy that accounted for repeated measures. Furthermore, while our sample is national in scope, there are likely some SSPs operating without legal authorization that were not included in our sample that might influence the findings regarding CBOs without funding. However, this would have not changed the overall finding regarding DPH SSPs relative to CBOs. Relatedly, the organizational categories used in this study are convenient for broadly defining SSPs but provide no insight into the cultural differences that might exist as well as relationships with the community. As part of this, we recognise that DPH SSPs might be located within social-legal settings that are challenging when providing harm reduction services and while there are limitations in our approach, we attempted to capture this context by controlling for community level factors. These community-level confounders were selected a priori based on other analyses of the NSSSP data underway and are not exhaustive. There could be other factors influencing SSP implementation, but these are not likely influence our key findings regarding differences between CBOs and DPH SSPs. With these factors we use counties as a unit of analysis which represent geographic boundaries that do not correspond to populations either in terms of size or characteristics; and many public health decisions and programmes are implemented at a state-level and not at the county level. However, as it regards SSPs, states, including states that have more recently allowed SSPs, have left the final decision and the implementation of SSPs to county and local governments.^35^ Additionally, it is possible that recent SSP implementations are more likely to be DPH SSPs compared to CBO SSPs, and as earlier implementations, these newer programmes would likely be lower scale, on average. Finally, the current study is unable to speak to the impact of harm reduction efforts among SSPs in reducing overdose mortality, nor disparities in those mortality rates.

### Conclusion

SSPs have become multipurposed, providing resources for a variety of healthcare needs, but the history of these efforts is rooted in community-led responses from, and for, persons who use drugs. With the United States government now providing some funding for SSPs there is a need for further research aimed at understanding how organizational context influence the optimal delivery of services within specific policy contexts. Based on this study, government funded CBOs appear to be associated with expanded or sustained services in our findings and scaling this could be an effective public health strategy to nationally expand the role of SSPs beyond protecting communities from infectious disease outbreaks and serving as key setting for the implementation of harm reduction and overdose response services.

## Contributors

BRR conceptualized the study with JLH and BHL who, along with LDW and AHK contributed to funding acquisition. BRR drafted the initial manuscript and managed revisions with SVP, CFA, RNB, HT, and PL who provided additional guidance, review, and/or drafted portions of the manuscript. JLH and BHL has access to raw data, verified the data and analysis, and BRR was responsible for the final decision to submit the manuscript. All authors read and approved the final manuscript.

## Data sharing statement

The 2020 Census, National Centre for Health Statistics Urban-Rural Classification Scheme, and MIT Election data are publicly available; however, given concerns about reidentification the National Survey of Syringe Services Programs is not available.

## Declaration of interests

BRR reported funding from the National Institute on Drug Abuse and the Centres for Disease Control and Prevention for projects unrelated to this work. RNB has received honoraria from the University of California Global Health Institute, the University of California Los Angeles, and the University of Kentucky, serves on the Council for Black Health, and reported funding from the National Institute on Drug Abuse and Robert Wood Johnson Foundation for projects unrelated to this work. HT serves on the board of the SAFE Project and HIVMA, grants or contracts from Gilead Sciences and Viiv, and consulting fees from the National Institute on Drug Abuse and the Centers for Disease Control and Prevention for projects unrelated to this work. BHL reported funding from the National Institute on Drug Abuse for projects unrelated to this work and funding from Arnold Ventures that supported this study.
